# Ten Inventions That Shaped Modern Orthopedics

**DOI:** 10.7759/cureus.12819

**Published:** 2021-01-20

**Authors:** MN Baig, SR Kearns, Fintan J Shannon, A Devitt

**Affiliations:** 1 Trauma & Orthopaedics, University Hospital Galway, Galway, IRL

**Keywords:** arthroplasty, external fixator, bone cement, pedicle screw

## Abstract

The current field of orthopedics is the result of many decades of minor and major advancements. The evolution of orthopedics has culminated into the modern field seen today. This article presents 10 inventions that played a key role in shaping modern orthopedics.

## Introduction and background

Some of the most common phrases heard in medicine speak to the state of advancement of modern medicine: “We are now on the threshold" or “This is on the cutting edge.” Our cutting edge is no sharper today than it has been over the centuries with every new major invention. Human history is full of landmark surgical and medical inventions. This review describes recent important orthopedic inventions that have shaped the state of modern orthopedic practice. We describe innovations still in use in their original forms and some inventions that have improved over time with enhancements but retain their original principles. Hundreds of inventions helped the field of orthopedics become a successful modern specialty. This review, however, focuses on 10 inventions that provided the most far-reaching and influential impact.

Most ideas are not truly new ideas; external fixation is a typical example of an old idea still in use today. An early description of external fixation is found in Hippocrates’ writing, but even he is believed to have described methods of treatment used for centuries by his predecessors.

## Review

While hundreds of inventions have shaped the field of orthopedics as a modern specialty, this review focuses on 10 inventions that have had the most considerable impact on modern orthopedic medicine.

X-rays

**Figure 1:**
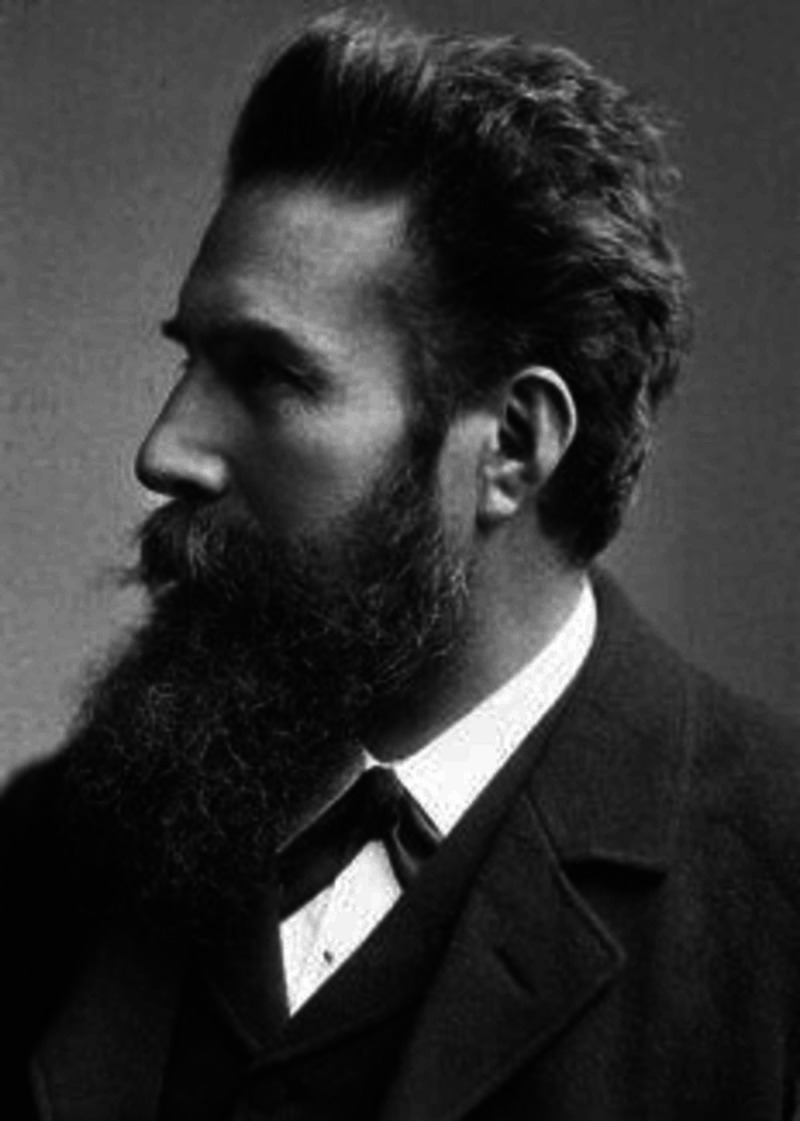
Wilhelm Conrad Röntgen Courtesy: https://www.nobelprize.org/prizes/physics/1901/rontgen/biographical/

The discovery of the x-ray in 1895 was the most instrumental in the rapid advancement of biological sciences, especially in orthopedics [1]. Wilhelm Röntgen, professor of physics in Wurzburg, Bavaria, was the first person to discover the use of electromagnetic radiation to create the x-ray (Figure 1). Röntgen performed the first x-ray capturing the bones of his wife’s hand [2].

Röntgen was working on finding the path of the electric rays in an empty glass cathode ray tube (i.e., Crookes tube) covered with black paper. A green, fluorescent light could be seen coming from the tube. He found that the new ray would pass through most substances casting shadows of solid objects on pieces of the film. He named them x-rays, as, in mathematics, the unknown quantity is named “x.” He received the Nobel Prize in Physics in 1901 for his discovery [2].

**Figure 2:**
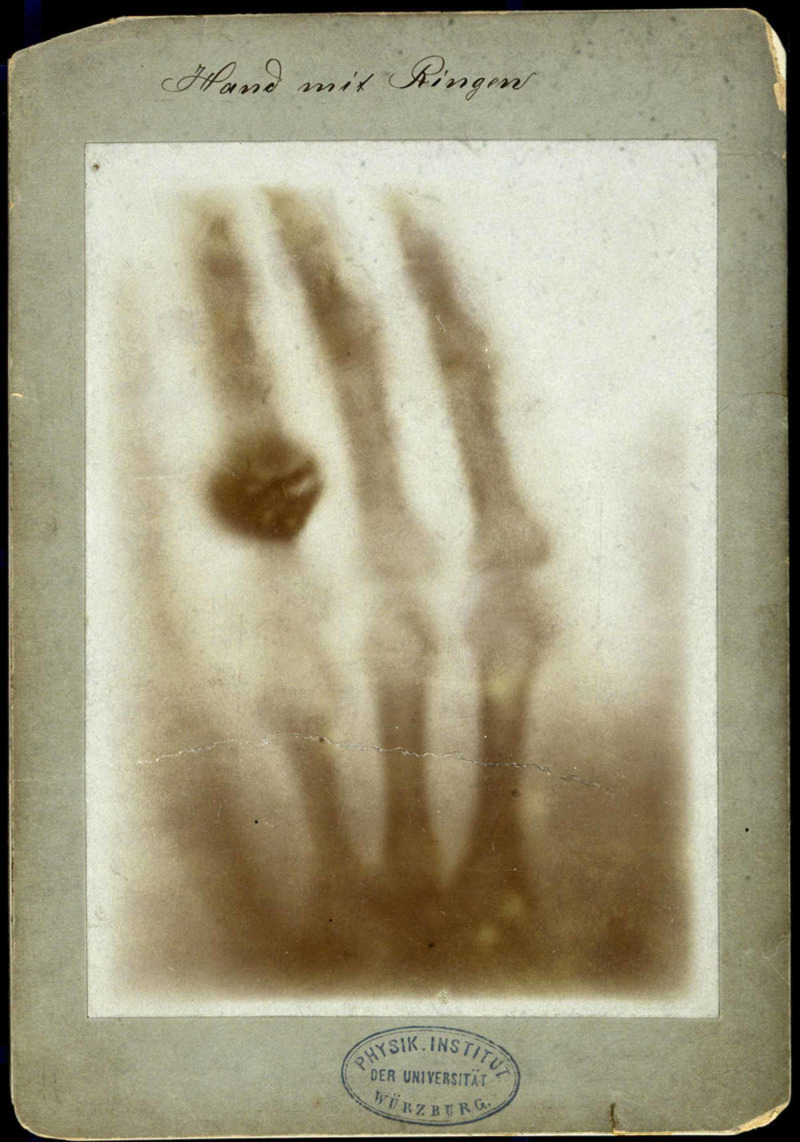
The first x-ray image, “Hand mit Ringen” Courtesy: https://www.atlasobscura.com/articles/roentgen-xrays-discovery-radiographs

Performing an x-ray was tedious, according to the experience of a modern medical physicist at the Maastricht University Medical Center in the Netherlands, Gerrit Kemerink, who discovered an x-ray machine from the 1890s and tried to obtain an x-ray of a hand (Figure 2) [2]. He found that the original machine used 1500 times the radiation used in modern radiology methods. Moreover, it took him 90 minutes to obtain an x-ray with the 1890s machine compared to the 20 ms needed for a modern x-ray machine.

The discovery of the x-ray was the most instrumental in the rapid advancement of biological sciences, especially in orthopedics.

Bone cement

**Figure 3:**
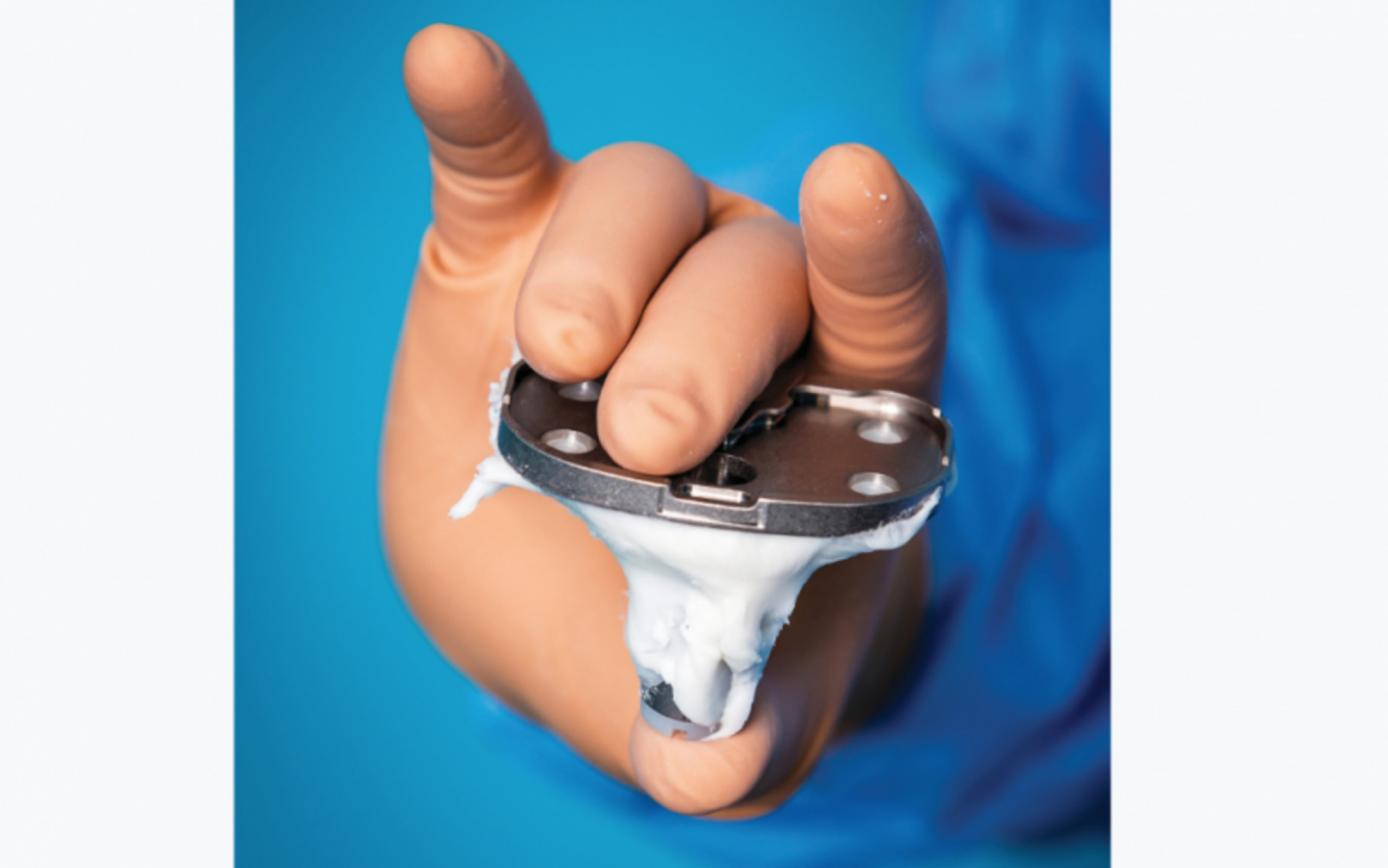
Bone cement around tibial implant (total knee replacement) Courtesy: http://www.opnews.com/2020/01/onset-of-a-minimally-invasive-era-to-set-new-possibilities-for-the-bone-cement-industry/15974

Themistocles Gluck (1870) used cement made of plaster and colophony for his knee arthroplasty technique [3]. John Charnley, the English surgeon famous for his use of low-friction arthroplasty in 1958, used bone cement for total hip arthroplasty. He used cold-cured polymethyl methacrylate to attach an acrylic cup to the femoral head and seat a metallic femoral prosthesis [3]. In reality, “cement” is a misnomer because cement is used to describe a substance that bonds two things together. However, bone cement acts as a space-filler that creates a tight space that holds the implant against the bone and acts more like a “grout” (Figure 3). It is mainly used in joint arthroplasty, vertebroplasty, and kyphoplasty [4].

Low-friction arthroplasty

**Figure 4:**
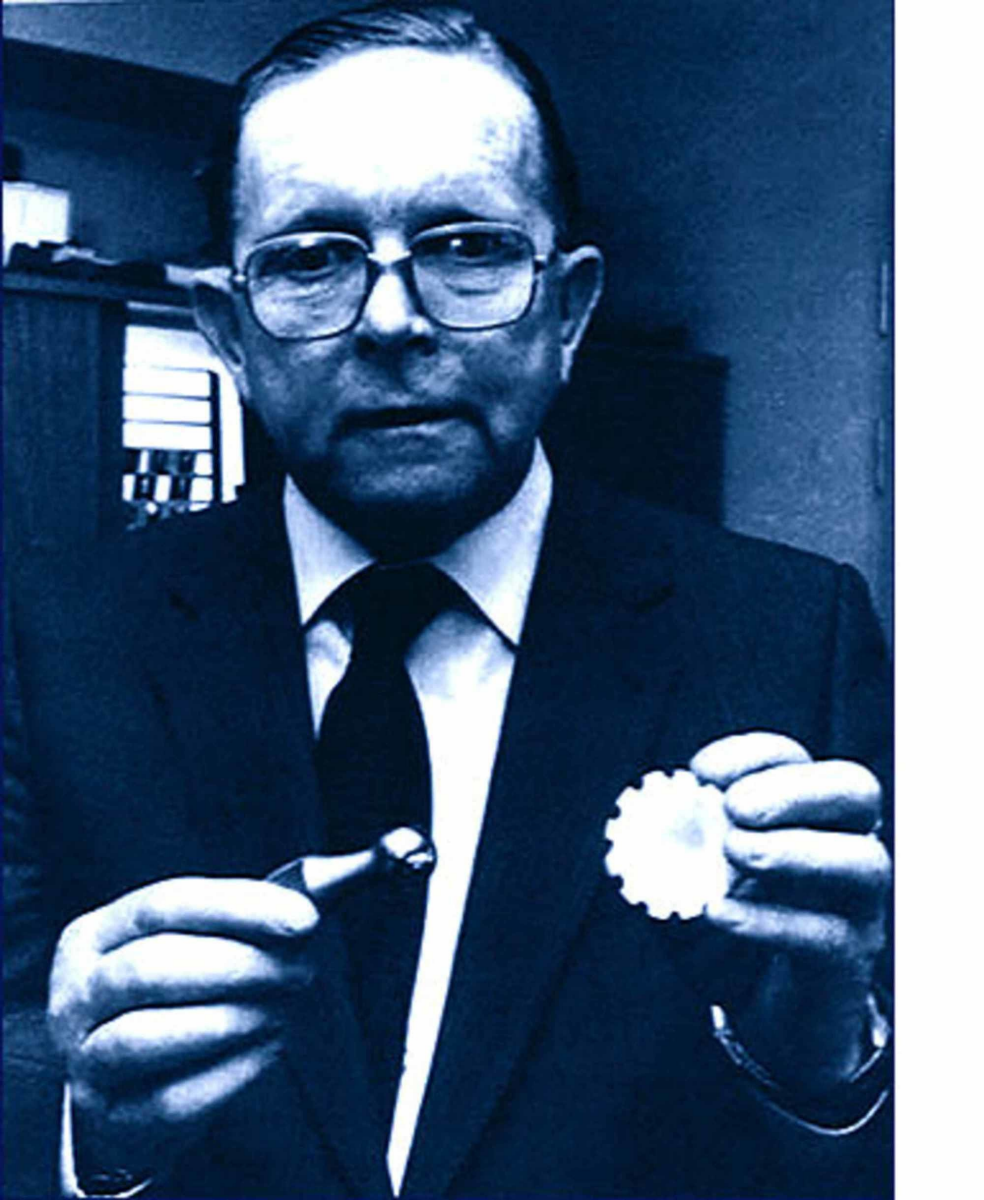
John Charnley Courtesy: http://www.johncharnleytrust.org/awards.html

In November 1962, John Charnley (Figure 4), an orthopedic surgeon at Wrightington Hospital, developed a technique that started a successful arthroplasty era in orthopedics [5]. Hip arthroplasty had been going on before his time, but he pioneered the concept and practice of successful low-friction arthroplasty [6].

**Figure 5:**
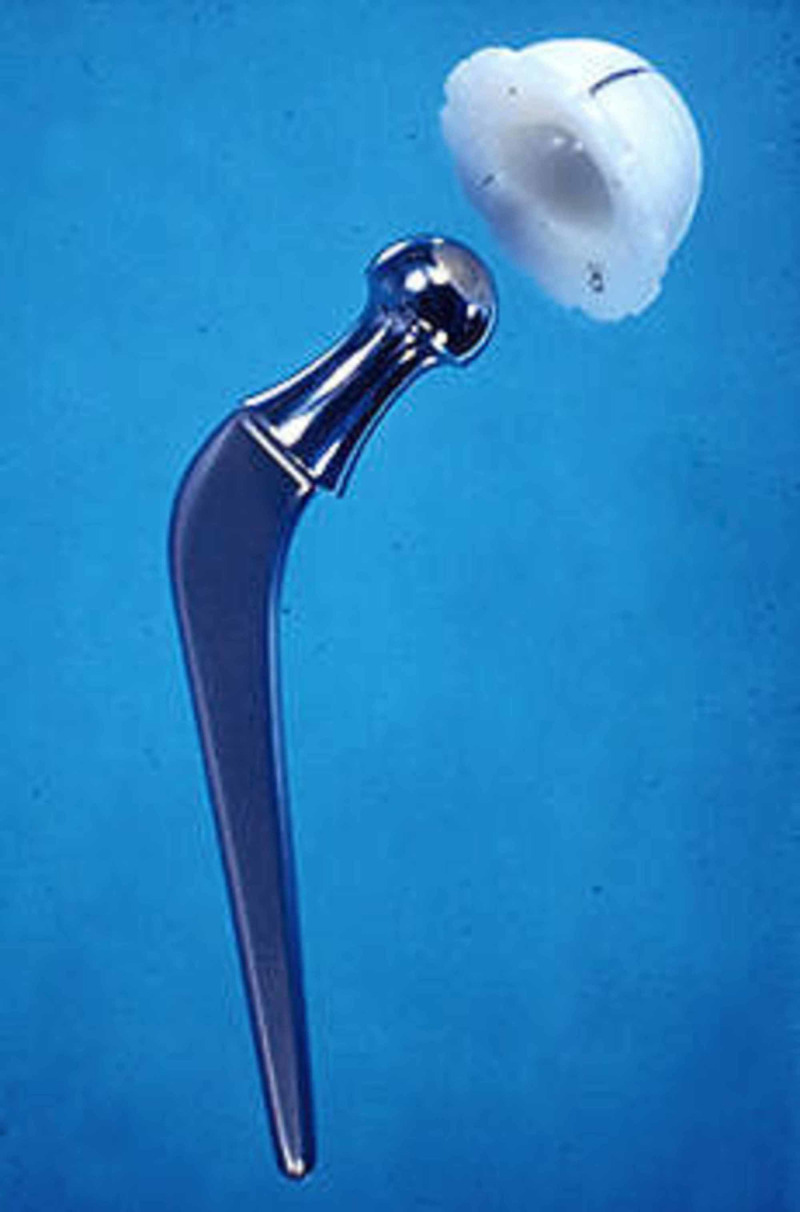
Charnley low friction arthroplasty Courtesy: http://www.luigigentilemd.com/HipKnee/THE%20CHARNLEY%20TOTAL%20HIP%20REPLACEMENT.htm

Charnley concentrated on reducing friction in arthroplasty; he considered friction to be the reason for arthroplasty failures. As Charnley said, “The cart has been put before the horse; the artificial joint has been made and used, and now we are trying to find out how and why it fails” [6]. He sought to develop a material that would minimize friction between the femur head and the acetabular shell. He used polytetrafluorethylene (i.e., Teflon) in 1956 as a synthetic cartilage replacement, but its wear and intense tissue reaction presented significant challenges. He then discovered ultra-high molecular weight polyethylene, which was successful due to its strength, low wear rate, and low friction. Charnley also improved his technique by working on reducing the turning force (torque) of the metal head on the acetabular component. He achieved this by bringing the femoral head size to 22.2 mm. The changes in materials and the design principle comprise the landmark Charnley concept (Figure 5) [5].

Intramedullary nailing

Historians have suggested that as early as the 16th century, the Aztecs placed wooden sticks as braces for long bone fractures. In the modern era, Swiss-born American surgeon Nicholas Senn, in 1893, was the first surgeon to conceptualize and work on intramedullary (IM) fixation of long bones [7]. Many surgeons after him furthered this practice, such as Themistocles Gluck, Nicolaysen, the Rush brothers, and Ernest William Hey Groves [7].

**Figure 6:**
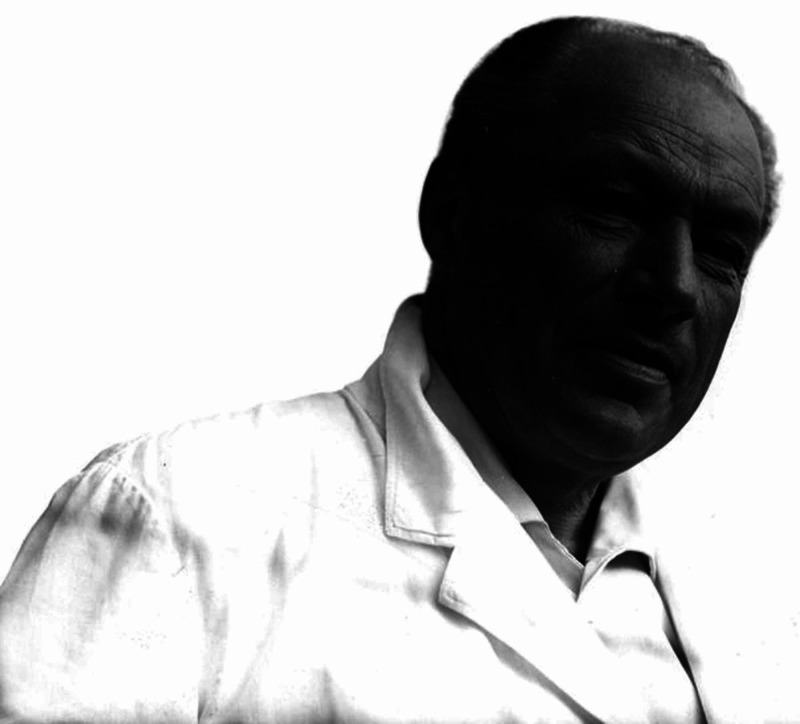
Gerhard Küntscher Courtesy: https://musculoskeletalkey.com/history-of-intramedullary-nailing/

The next leap in IM fixation was made by a German surgeon, born in 1900, named Gerhard Küntscher in the form of “Marrow nails” (Figure 6). He developed the principles of IM nailing that used an entry point of nails away from the fracture site, with an IM device applied to the full length of the long bone, and emphasized the stability of the fixation and its relation to the diameter of the device. Küntscher’s first nailing procedure on a human was performed in 1939 [7,8].

**Figure 7:**
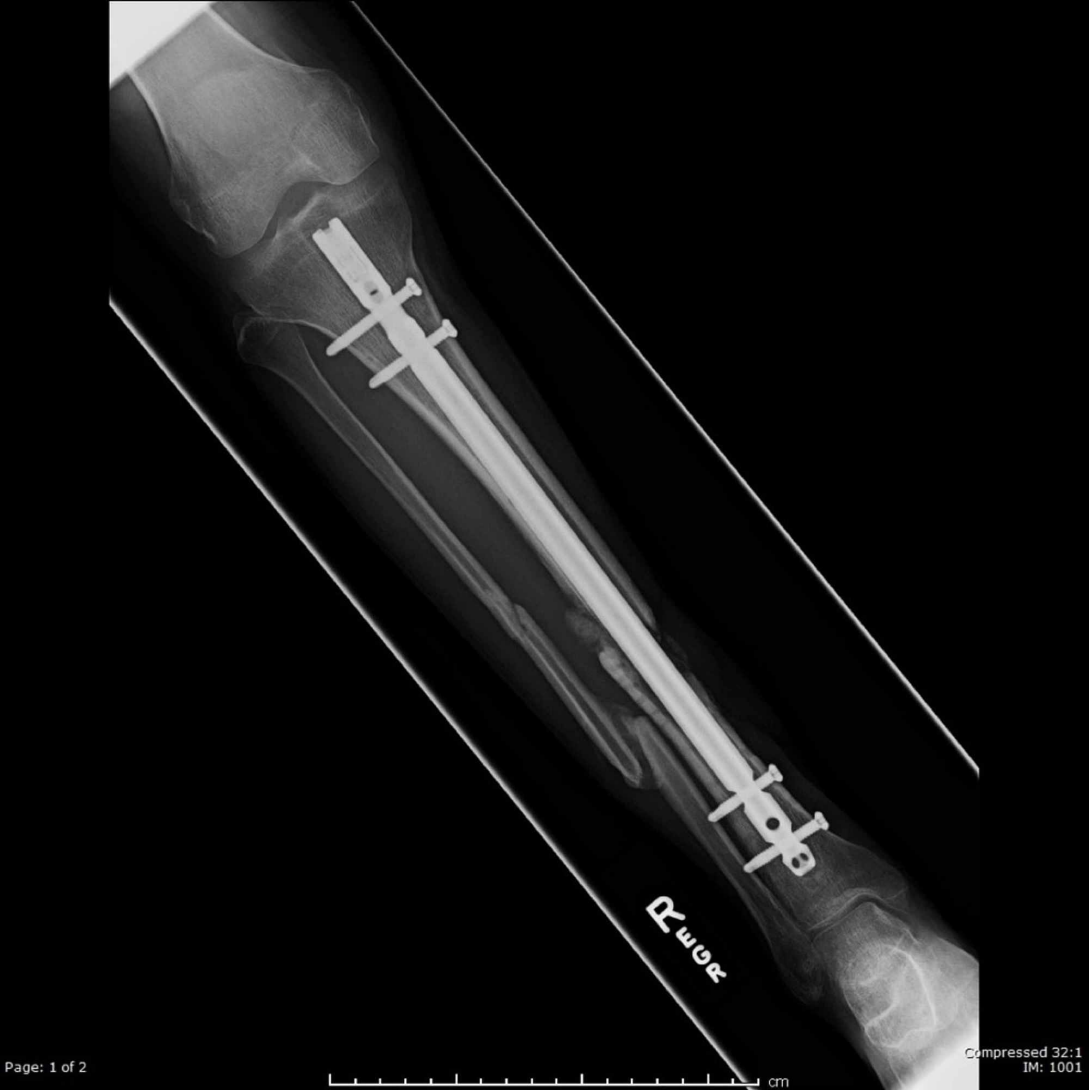
An intramedullary nail Courtesy: https://radiopaedia.org/cases/intramedullary-nailing-and-reaming-irrigator-aspiration-ria-autografting-1

The technique and implant of IM nailing have improved drastically. Different types of nails have been developed, and they have been classified into four generations of IM nails (Figure 7) [8].

Thomas splint

**Figure 8:**
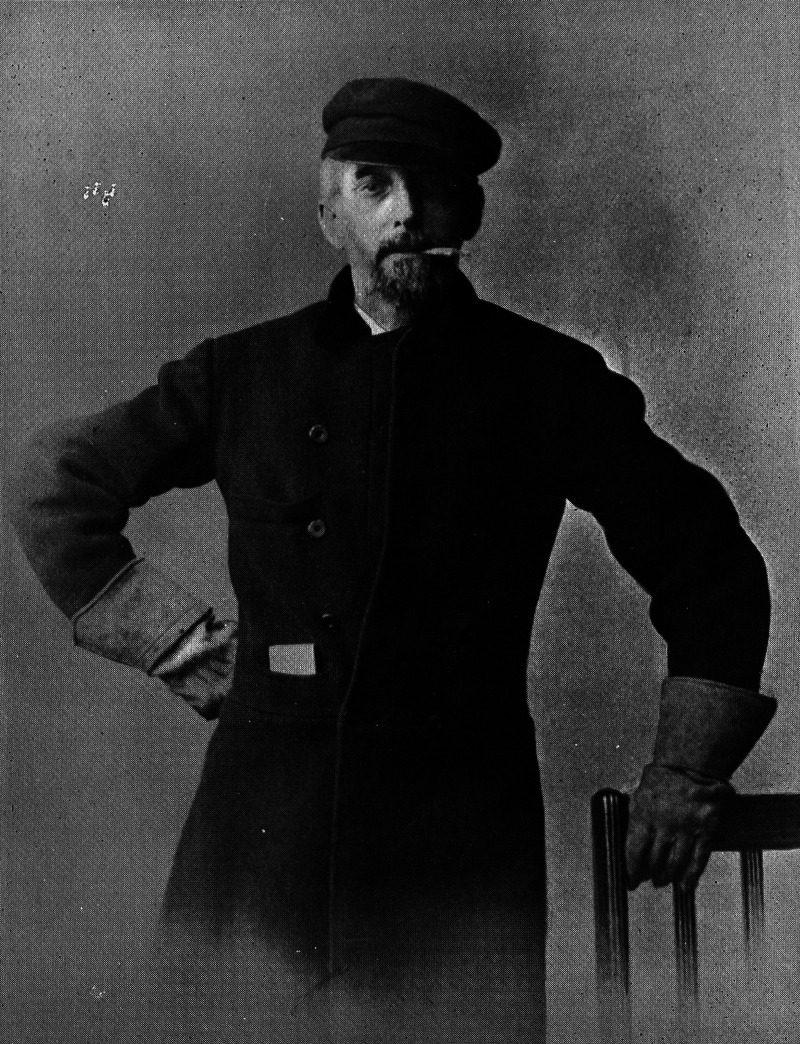
Hugh Owen Thomas Courtesy: https://wellcomecollection.org/works/x8hf9mv3

The Thomas splint was designed and used for the first time by Hugh Owen Thomas from Bodedern, North Wales, UK (Figure 8). The Thomas splint is a basic and essential device commonly used in emergency departments worldwide. Thomas described the concept and design of the splint in 1876 [9].

**Figure 9:**
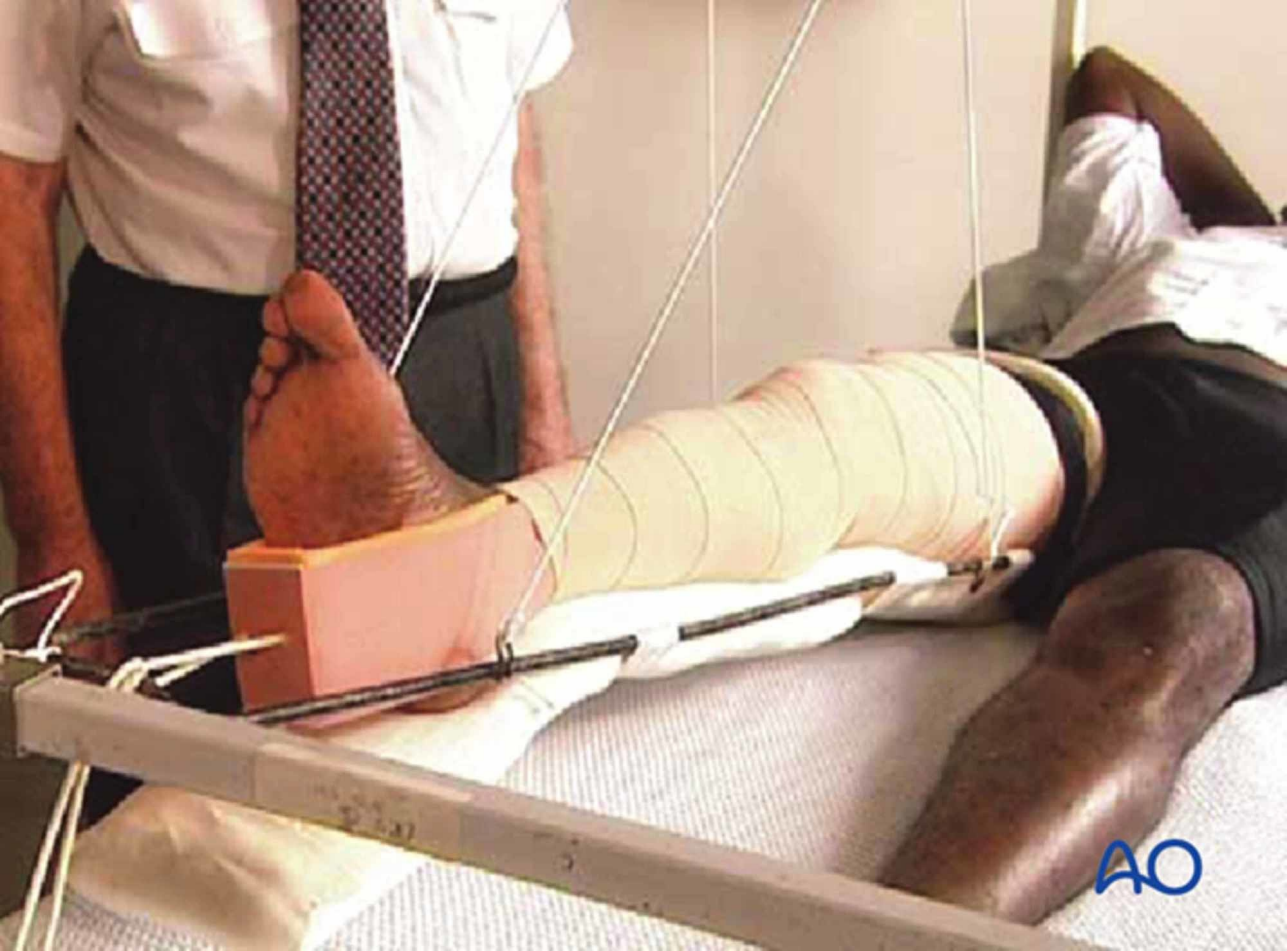
The Thomas splint Courtesy: https://surgeryreference.aofoundation.org/orthopedic-trauma/adult-trauma/femoral-shaft/wedge-fragmentary-middle-1-3-fractures/temporary-thomas-splint

Before the advent of the Thomas splint, bone injuries carried a high mortality rate, especially in wartime due to inferior splintage methods and inadequate treatments. Sir Robert Jones, the nephew of Thomas, was a significant figure in popularizing the Thomas splint for stabilizing the femur and tibia during the First World War. Before the Thomas splint’s advent, amputation was considered the better treatment for femoral fractures for saving lives. Sir Henry Gray, a British military surgeon, reported in 1917 that the mortality rate for femoral fractures dropped from 80% to 15.6%. The splint was popular due to its simple design, ease of use, and effectiveness in stabilizing long bone fractures (Figure 9) [10].

External fixator

Hippocrates described a technique of external fixation using leather and wooden sticks for anatomical alignment and maintaining length. In the modern era, the external fixator is a device used to treat fractures either permanently or as a standby arrangement to avoid further damage until the permanent treatment can be applied.

External fixator use was popularized in the mid to late 1800s when many devices were used as external fixators. Jean-Francois Malgaigne, Clayton Parkhill, and Lambotte used it, and it evolved in both efficacy and design [11].

**Figure 10:**
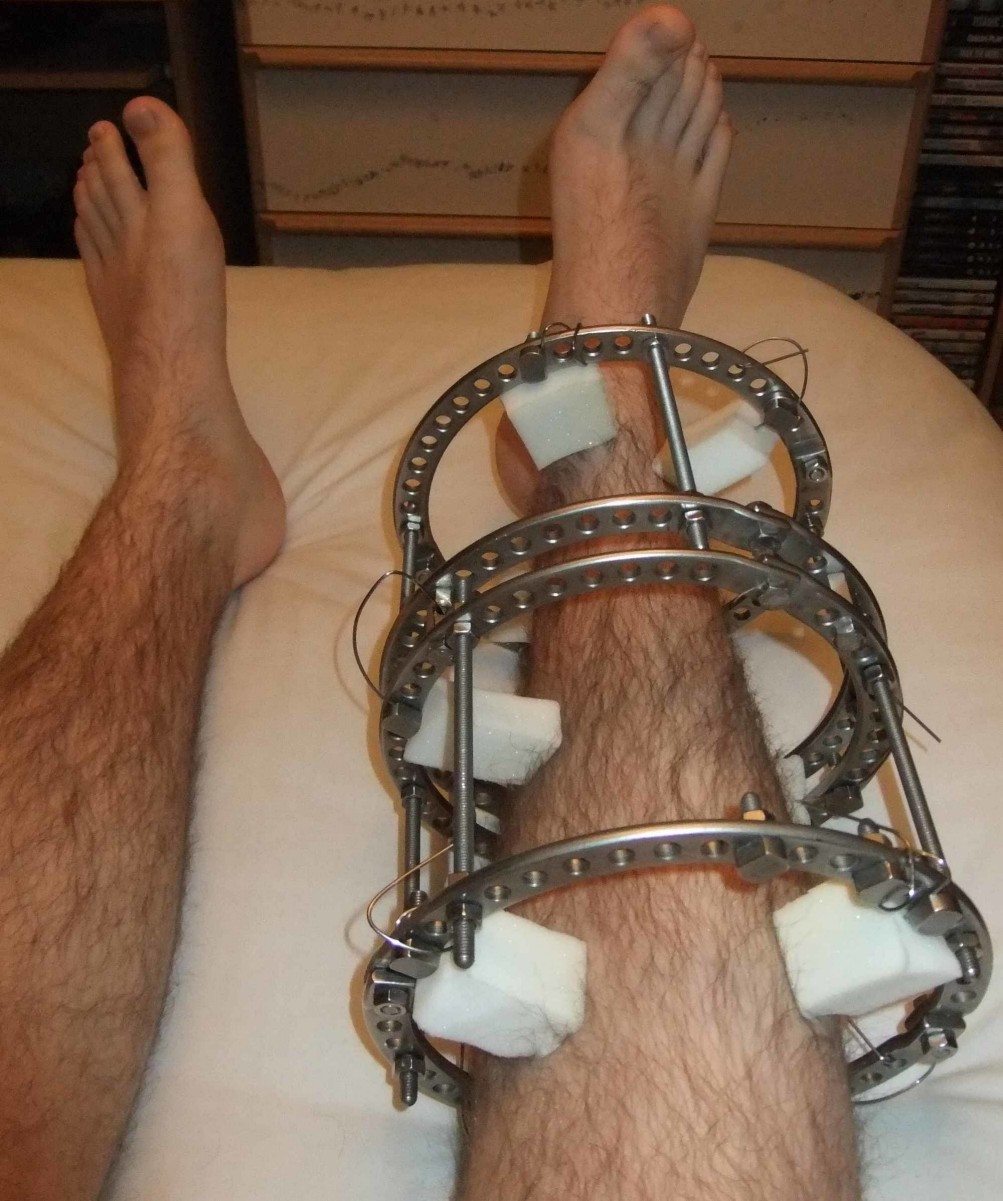
Circular External Fixator Courtesy: https://en.wikipedia.org/wiki/External_fixation

In 1938, Swiss doctor Raoul Hoffmann described and used a technique of closed reduction with percutaneous pinning. Behind the Soviet Iron Curtain in Kurgan, Siberia, Professor Gavriil Ilizarov designed a new method of external fixation in the 1950s and used it for traumatic injuries, limb lengthening, correction of congenital limb deformities, bone defects, osteomyelitis, and other bone deformities (Figure 10) [12].

In 1989, Behrens described three simple and straightforward principles of use for external fixation. According to these principles, an external fixator should protect vital structures when pinning, allow access to the area of injury, and be biomechanically sufficient to treat the fracture.

**Figure 11:**
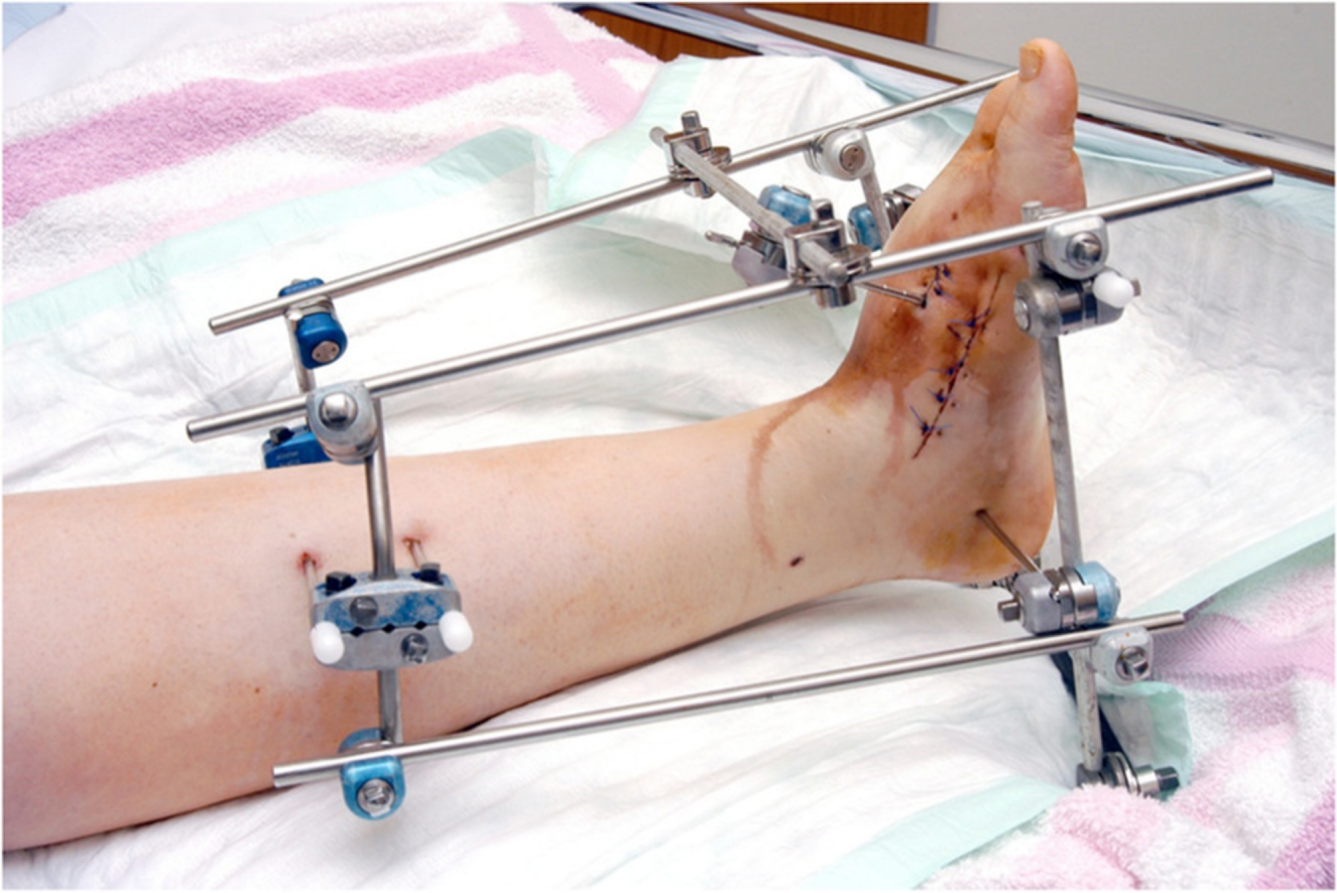
Double-frame external fixator https://www.researchgate.net/publication/268789076_Clinical_benefit_and_improvement_of_activity_level_after_reconstruction_surgery_of_Charcot_feet_using_external_fixation_24-months_results_of_292_feet/figures?lo=1

Today, external fixators are used regularly as a mainstay treatment and as a part of makeshift treatment during damage control situations. Commonly used current external fixators are uniplanar, biplanar, and multiplanar (i.e., circular) fixators (Figures 10, 11) [12].

Magnetic resonance imaging

Magnetic resonance imaging (MRI) is a relatively new imaging technique. Felix Bloch and Edward Purcell independently discovered the basic principle of MRI, the nuclear magnetic resonance phenomena, in 1946. They were awarded the Noble Prize in 1952. Early in its use, MRI was considered part of chemical and physical analysis. In 1971, Raymond Damadian showed that this new technology could detect different diseases, including tumors, in humans [13].

Paul Lauterbur at Stony Brook University and Peter Mansfield at the University of Nottingham (England) furthered the use of MRI by applying it to human diagnostic use in the 1970s. They were awarded the Noble Prize in 2003 for physiology and medicine [14].

**Figure 12:**
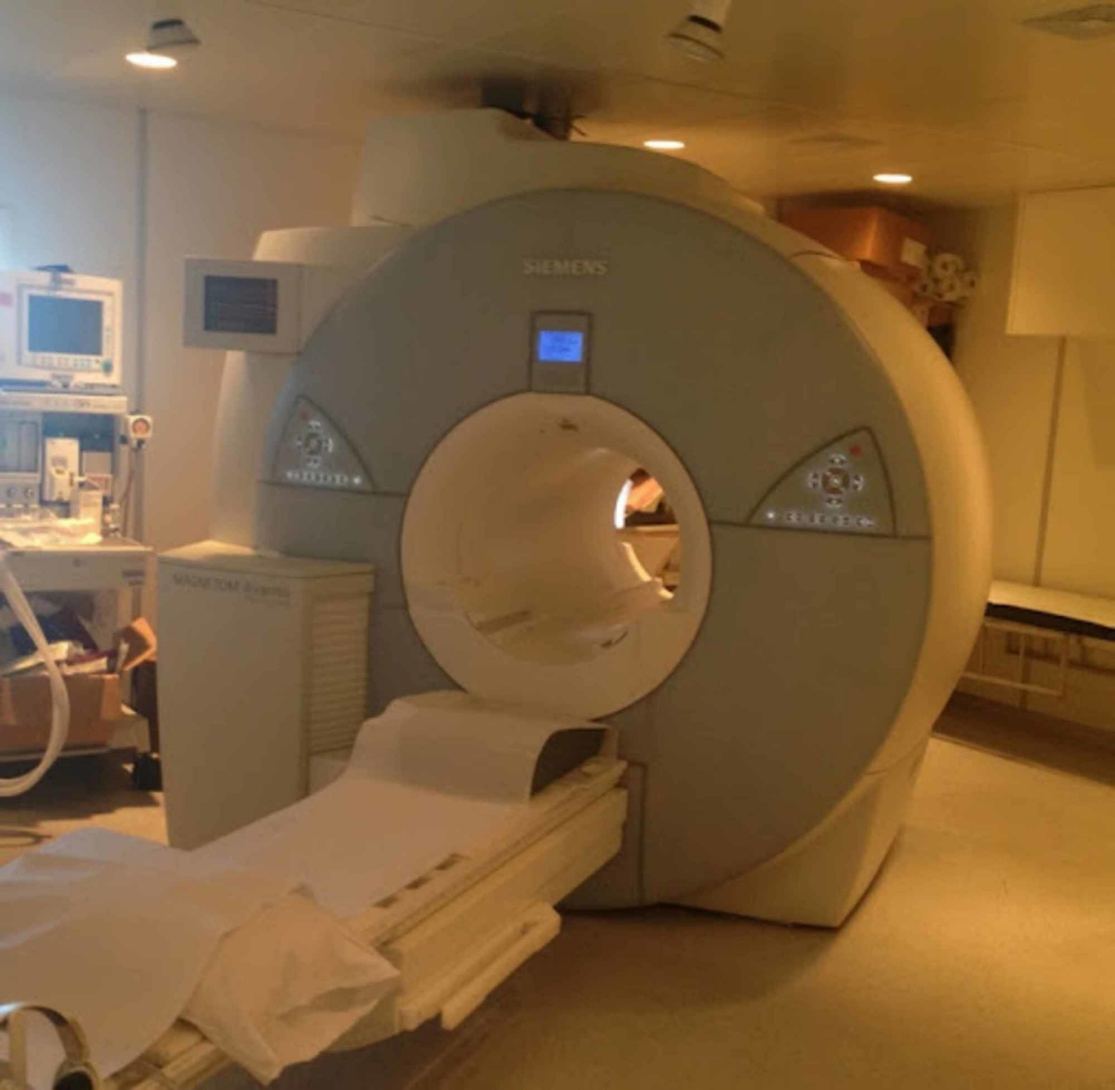
A magnetic resonance imaging scanner Courtesy: http://www.svuhradiology.ie/diagnostic-imaging/mri/

From 1977 to 1978, the first MRI machine was built in Downstate Medical Center, New York. Modern MRI machines use superconducting magnets and radiofrequency coils to orient protons, resulting in image production (Figure 12). There are multiple MRI sequences, but the common sequences are T1-weighted, T2 -weighted, short tau inversion recovery, spin echo, and fat-suppressed MRIs [13].

Initially, MRI was called nuclear magnetic resonance, but then “nuclear” was dropped to prevent people from worrying that it had something to do with radioactive substances, which it did not.

MRI includes no ionizing radiation exposure, and it allows for the acquisition of multiplane images without repositioning the patient and better soft tissue imaging. MRI has two essential disadvantages: it is expensive and unsafe for patients with some metal implants.

Reverse shoulder arthroplasty

**Figure 13:**
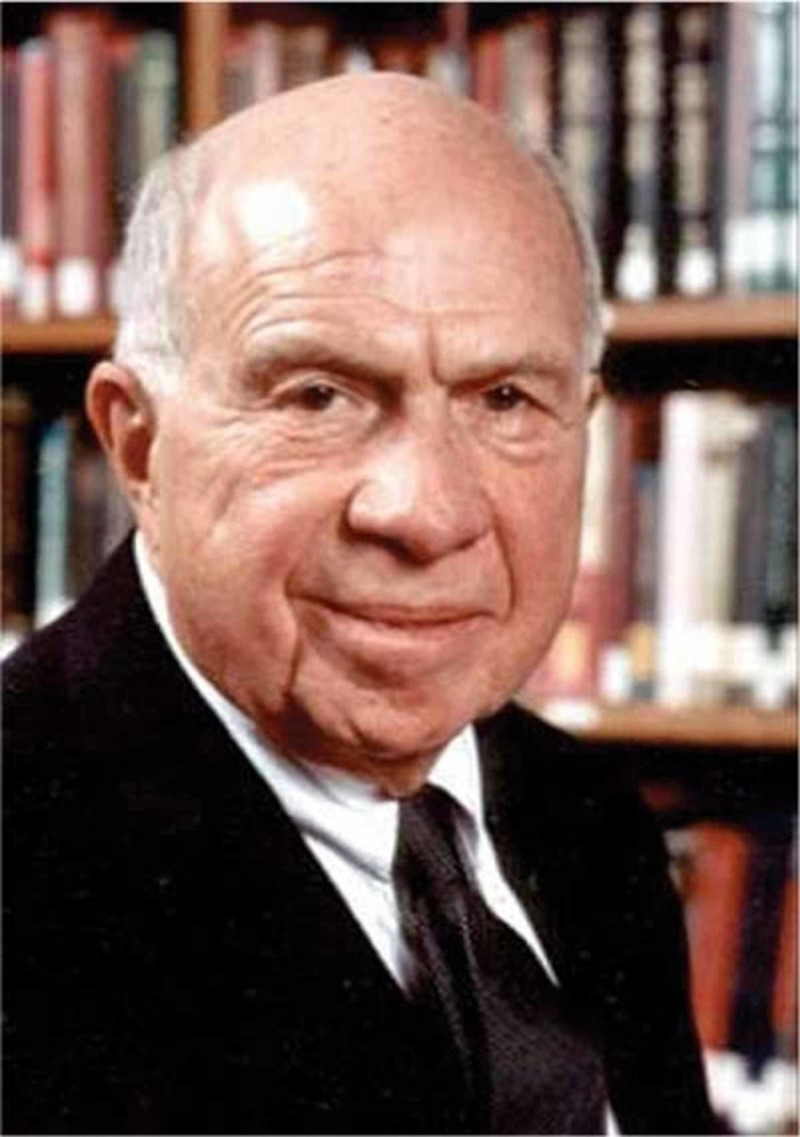
Charles Neer Courtesy: https://musculoskeletalkey.com/treating-the-rotator-cuff-deficient-shoulder-the-columbia-university-experience/

Jules-Émile Péan, a French surgeon, performed the first shoulder arthroplasty. Charles S. Neer, a professor of orthopedic surgery at Columbia University, is considered the father of shoulder arthroplasty (Figure 13). He improved on the shoulder arthroplasty design and implant [15].

The complications in routine shoulder arthroplasty had been the loosening of the glenoid component; the surgeons started to use cement, pegs, and screws to fix the glenoid component. However, due to limited glenoid bone stock, another complication arose in scapular/glenoid fractures. To address this, surgeons in the 1970s experimented with reversing the anatomy of the arthroplasty components. The advantages of this design were a better range of motion and less risk of dislocation/fracture. The first major reverse shoulder arthroplasty system was the Leeds/Reeves system in 1972 [16]. Over time, the process improved, and many other prosthesis systems were introduced, including the Kessel (1973) and Bayley-Walker (1973) prostheses [16].

In 1985, Paul Grammont devised a new concept of medializing and lowering the center of rotation in the new prosthesis design, emphasizing deltoid function. In 1991, the second version of Grammont’s design (the Delta 3) with further medialization was developed. In 1994, the third-generation Grammont design was developed with additional improvements [16].

The design and implants have been improved over the years, but they still follow Grammont’s principles, which required that the prosthesis be inherently stable, the weight-bearing surface should be convex, the center of the sphere should be in glenoid, and the center of rotation should be medialized.

**Figure 14:**
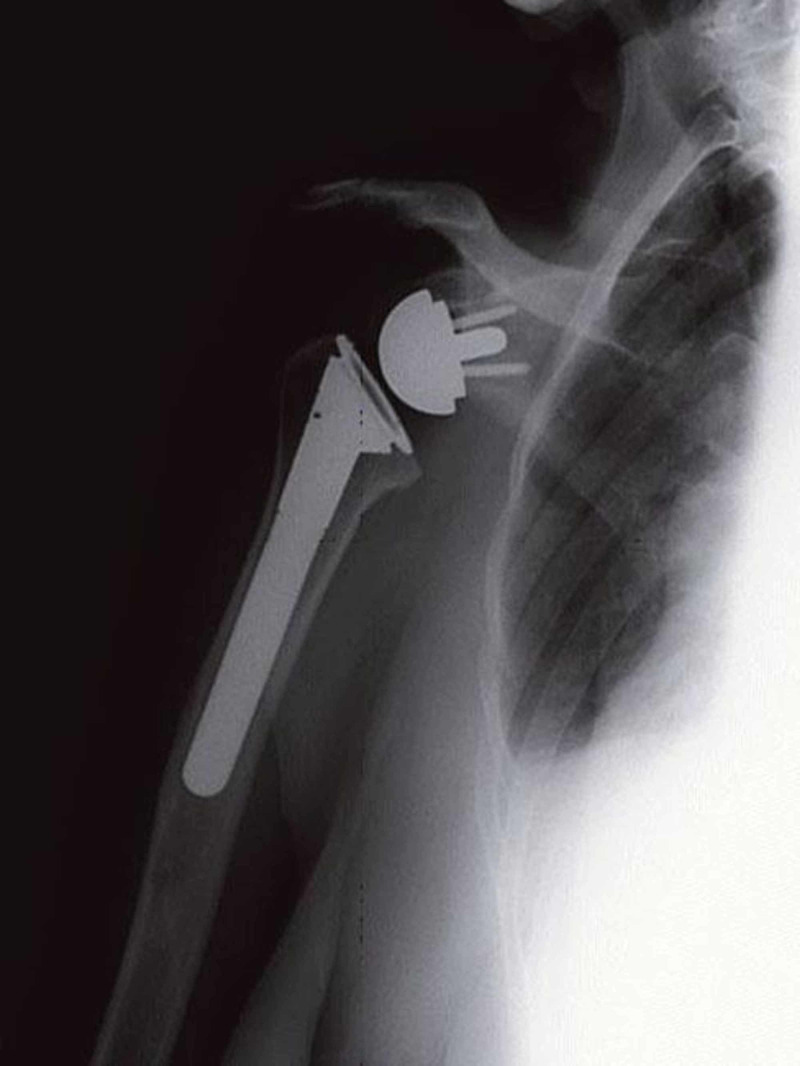
Reverse shoulder arthroplasty Courtesy: https://musculoskeletalkey.com/treating-the-rotator-cuff-deficient-shoulder-the-columbia-university-experience/

The reverse shoulder arthroplasty is used mainly for cuff tear arthroplasty, pseudoparalysis, failed conventional arthroplasty failure, and rotator cuff deficiency conditions in low-functioning elderly patients with deltoid and glenoid bone stock intact (Figure 14).

Pedicle screw

The pedicle screw is an important innovation in spinal surgery. Boucher (1959) and Roy-Camille (1963) are credited with pioneering the use of vertebral pedicle screws [17]. At first, pedicle screws were used for traumatic fractures, but eventually, they were used for a broader set of conditions such as vertebral malunions, spinal deformity, spondylolisthesis, and tumors. Later, Louis and Maresca in Marseille, France, in the 1970s improved the pedicle screw and plate fixation method, the report of which in the literature popularized the use of pedicle screws [17].

**Figure 15:**
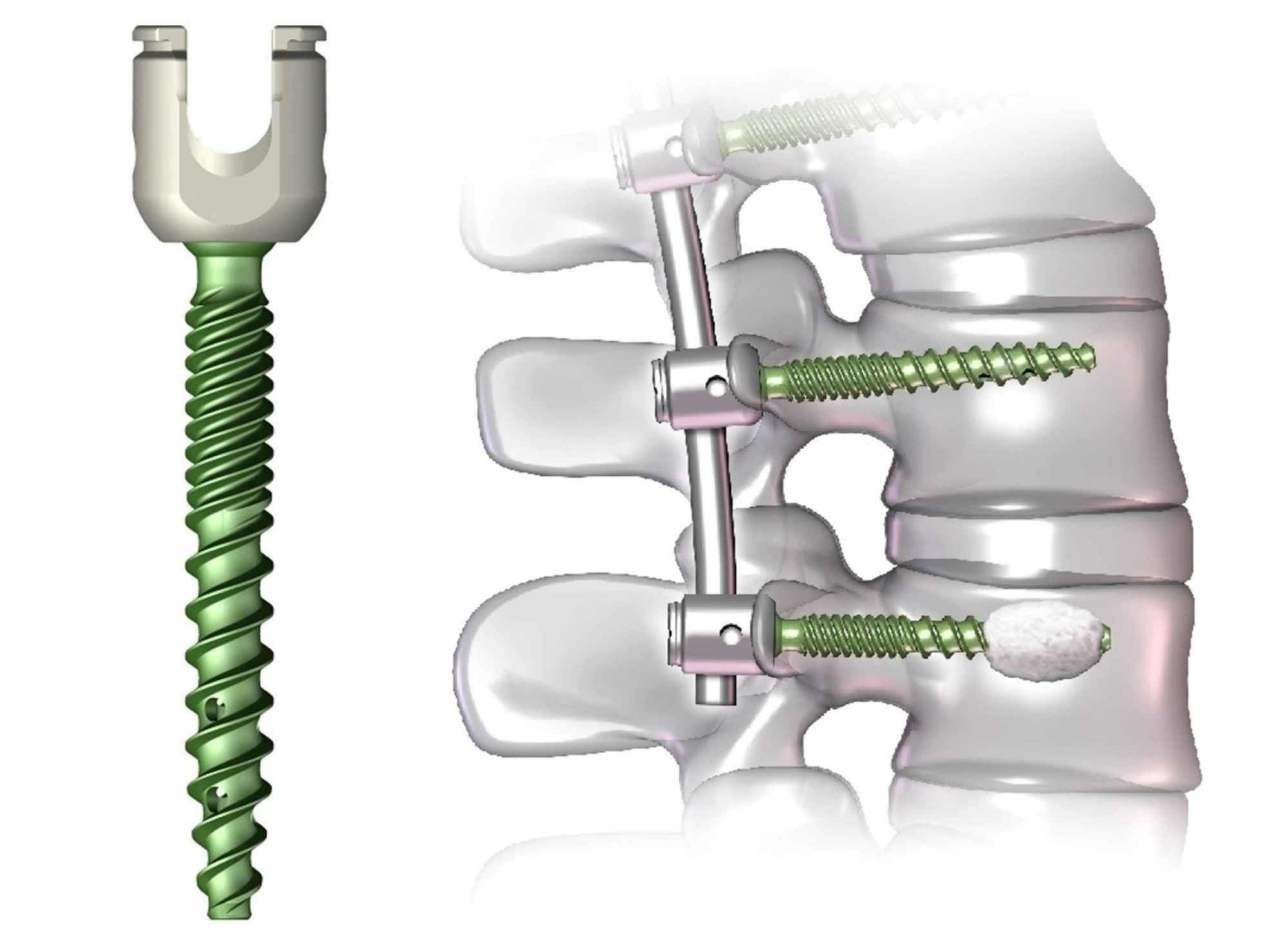
Pedicle screw Courtesy: https://spinalnewsinternational.com/depuy-synthes-receives-us-fda-clearance-for-cement-augmented-pedicle-screw-systems/

The principal of pedicle screw use is based on the rationale that the pedicle is the most robust site approachable posteriorly. The pedicle screws travel through all three vertebral columns, providing a three-dimensional positional control that is valuable in small segmental fixation (Figure 15) [18].

Another distinctive feature of pedicle screws is they do not need posterior elements to be intact to work. Even after laminectomy or traumatic injury to the spinous process or lamina, pedicle screws can be used. Pedicle screw use is associated with a reasonable fusion rate, less postoperative bracing/support, and less analgesic requirement than procedures that do not use pedicle screws.

Arthroscope

According to Marcel Proust, the “real voyage of discovery consists not in seeking new landscapes, but in having new eyes.” Arthroscopy is another major invention in orthopedics that changed surgery from open surgical procedures to minimal, keyhole surgery.

Professor Kenji Takagi from Japan used a cystoscope (used by urologists since 1878, designed by Maximilian Carl-Friedrich Nitze, a German urologist and pioneer in this field) in 1918 for the first time in orthopedics. Tuberculosis was prevalent at that time, and he used the scope to examine a tuberculous patients knee. He worked to improve the design for use in arthroscopy [19]. His biggest challenge was the diameter of the scope’s optical cannula, which was 7.3 mm, which was challenging to use in a practical sense. In 1931, he was able to reduce the size to 3.5 mm. He also advised distending the knee with fluid for better visual appreciation.

Another milestone in arthroscopy was when a Swiss surgeon, Eugen Bircher, modified a laparo-thoracoscope for orthopedic purposes in 1921.

**Figure 16:**
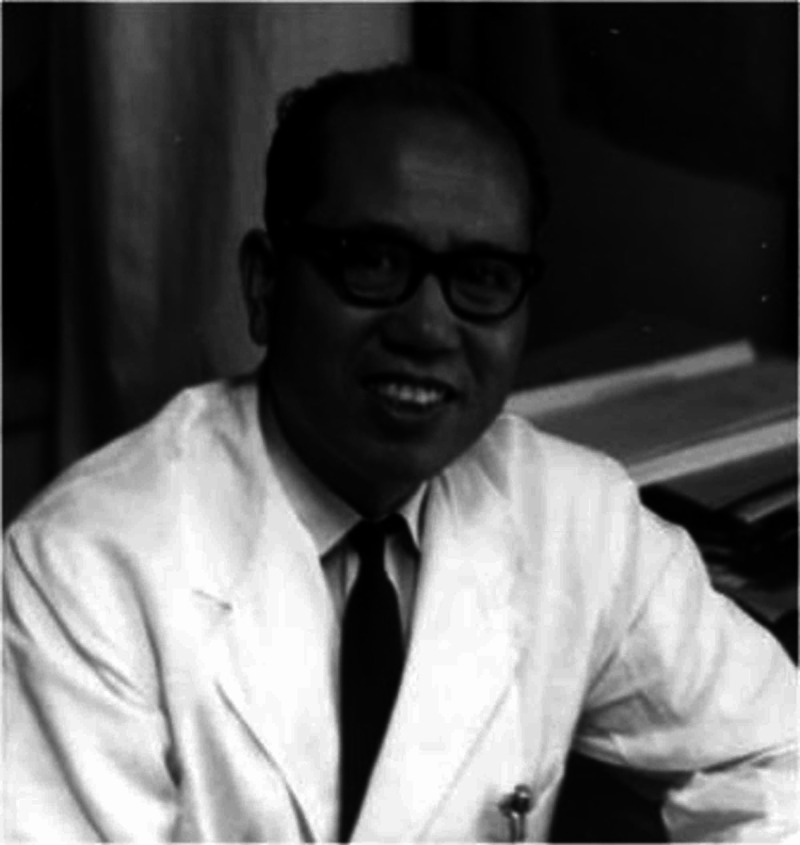
Dr. Masaki Watanabe Courtesy: https://www.sicot.org/enewsletter-94-history-orthopaedics

**Figure 17:**
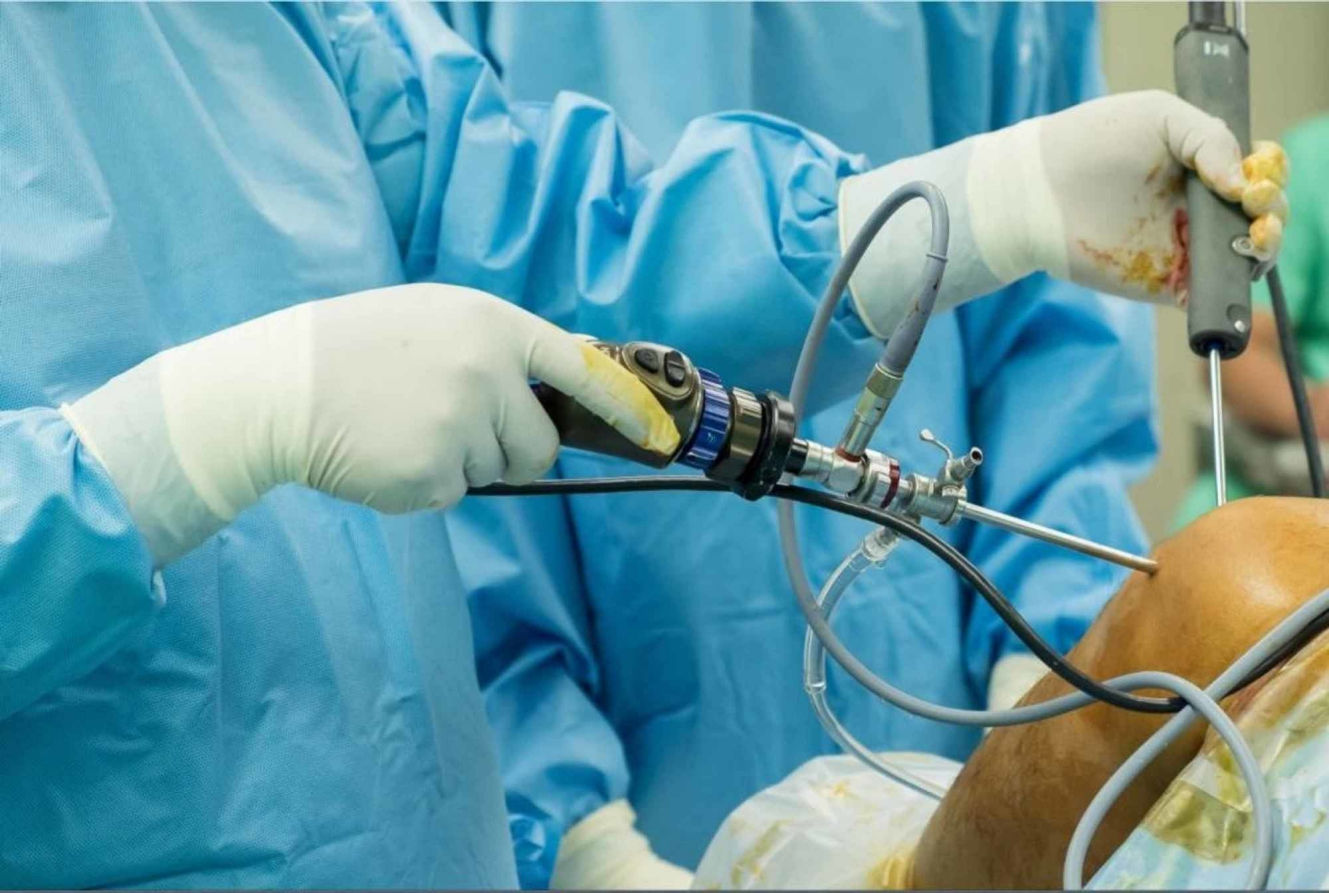
An arthrosope Courtesy: http://www.completesportscare.com.au/2019/10/knee-arthroscopy

Dr. Masaki Watanabe is known as the “father of arthroscopy” and continued working on the arthroscope’s design improvements (Figure 16). He introduced the concept of triangulation in arthroscopy [20]. He not only improved design, but he was also the first man to broaden the use of arthroscopy from a diagnostic tool to a therapeutic tool (Figure 17). In 1962, he performed the first arthroscopic meniscectomy on a 17-year-old boy.

## Conclusions

Knowing about these landmark inventions, the ideas behind their conception, and the people who invented them helps us by providing the perspective required to take on diagnostic and therapeutic challenges in the present. These inventions provide us with the understanding, the challenges, and the struggle of the inventors. They also provide the impetus for current and future generations of inventors.
